# Comparative Analysis of the Physicochemical Properties and Metabolites of Farinose and Crisp Lotus Roots (*Nelumbo nucifera* Gaertn.) with Different Geographical Origins

**DOI:** 10.3390/foods12132493

**Published:** 2023-06-27

**Authors:** Jiao Liu, Jiawen Peng, Jie Yang, Jing Wang, Xitian Peng, Wei Yan, Liuqing Zhao, Lijun Peng, Youxiang Zhou

**Affiliations:** 1 Hubei Key Laboratory of Nutritional Quality and Safety of Agro-Products, Institute of Quality Standard and Testing Technology for Agro-Products, Hubei Academy of Agricultural Sciences, Wuhan 430064, China; babojiao@126.com (J.L.); yangjie1127@163.com (J.Y.); yfzx1985@163.com (J.W.); pxitian@aliyun.com (X.P.); yanwei75126@163.com (W.Y.); nky_plj@163.com (L.P.); 2 Hubei International Scientific and Technological Cooperation Base of Traditional Fermented Foods, College of Food Science and Technology, Huazhong Agricultural University, Wuhan 430070, China; shjdbdjxveuxn@163.com; 3 SCIEX, Shanghai 200335, China; liuqing.zhao@sciex.com

**Keywords:** lotus root, texture, metabolites, Zeno trap, multivariate analysis

## Abstract

Lotus roots are widely consumed vegetables because of their great taste and abundant nutrients, but their quality varies with the environments and cultivar. This study systematically compared farinose (Elian No. 5) and crisp (Elian No. 6) lotus root cultivars from three geographical origins. Pasting and texture characteristics verified that Elian No. 5 possessed lower hardness and lower ability to withstand shear stress and heating during cooking compared with Elian No. 6. Untargeted metabolite profiling was first performed using ultrahigh-performance liquid chromatography coupled with electrospray ionization quadrupole time-of-flight mass spectrometry (UPLC-Q-TOF-MS) combined with a Zeno trap. In total, 188 metabolites were identified based on the matching chemistry database. Multivariate analysis demonstrated that lotus roots from different cultivars and origins could be adequately distinguished. Sixty-one differential metabolites were identified among three Elian No. 5 samples, and 28 were identified among three Elian No. 6 samples. Isoscopoletin, scopoletin, and paprazine were the most differential metabolites between Elian No. 5 and Elian No. 6. These results can inform future research on the discrimination and utilization of lotus roots.

## 1. Introduction

*Nelumbo nucifera* Gaertn., a perennial aquatic crop belonging to the family Nelumbonaceae, is consumed worldwide, including its rhizomes, seeds, leaves, flowers, and stamens [1,2]. Among them, the edible rhizome, also called the lotus root, is rich in starch, protein, vitamins, and some bioactive substances, such as beta carotene, polysaccharides, flavonoids, phenols, and triterpenes [3,4,5]. Coupled with its fascinating color, great taste, and potential antioxidant capacity, lotus root is accepted as a popular vegetable and fruit across the globe [6].

In recent decades, numerous studies have focused on increasing the shelf life of lotus root, and functional constituent analyses of lotus root have made good progress [7,8]. However, the nutrient concentrations in lotus roots vary among cultivars and are also affected by geographical locations, growing conditions, etc. [9]. To date, more than 300 kinds of lotus root cultivars are available from China and are distributed in the regions surrounding the mid-down Yangtse River, including Hubei, Jiangsu, Zhejiang, Anhui, Jiangxi, and Hunan provinces [2,10]. According to their taste and texture qualities, lotus roots can be broadly divided into farinose and crisp lotus roots. Generally, farinose lotus roots are cooked as braised dishes or in soup; in contrast, the crisp lotus roots are always cooked as stir-fry dishes or in salads. Based on this, there is great interest in the characterization of lotus roots treated by different cooking methods [11,12]. However, the characteristic compounds of lotus roots with different textures remain to be elucidated. Moreover, consumers pay more attention to food quality and the food source of origin [13], but the quality and characteristic of lotus roots from different regions are still ambiguous. Hence, the determination of lotus root cultivars and regions based on their characteristic substances is needed.

Metabolomics is an effective technology that can systematically identify and quantify small-molecule metabolites of a whole organism or tissue [14]. In addition, metabolomics in combination with multivariate statistical analysis is widely used in variety comparison and origin identification [15,16]. During the practice of metabolomics over the last 20 years, easier pretreatment, higher sensitivity, and more accurate identification are the core development directions of this technology [17]. Ultrahigh-performance liquid chromatography coupled with electrospray ionization quadrupole time-of-flight mass spectrometry (UPLC-Q-TOF-MS) is an effective tool for performing untargeted metabolomics analysis [18]. For instance, Yu et al. [19] identified the 101 chemicals including dihydromyricetin and flavonoids in lotus seeds. Guo et al. [20] determined 30 compounds consisting of flavonoids and alkaloids in lotus leaf extracts. Zhu et al. [21] characterized the 86 phenolic compounds in lotus seeds and rhizomes.

Recently, the application of Zeno trap pulsing technology in UPLC-Q-TOF-MS demonstrated an excellent ability to increase sensitivity, which can contribute to the accuracy of unknown compound identification [22,23,24]. Briefly, Zeno trap pulsing is a novel ion trapping/releasing strategy, and this technology can trap all fragment ions in the selected mass range in an axial pseudopotential well created by an additional radio frequency voltage. Then the ions are released by potential energy with timing aligned to the next pulse at the accelerator, thus increasing intensity and reducing the sample amounts needed. To the best of our knowledge, there is no report published on the metabolite digging of lotus roots by this approach.

In this study, typical farinose lotus roots (Elian No. 5) and crisp lotus roots (Elian No. 6) in China [25] were harvested from three cities of Hubei Province for comparative analysis. First, the pasting and texture properties of flours, starches, and slices from Elian No. 5 and Elian No. 6 were investigated to evaluate their texture differences. Then, a metabolomics approach based on UPLC-Q-TOF-MS in conjunction with Zeno trap pulsing was first employed to reveal the potential contributing ingredients. Multivariate analysis, including principal component analysis (PCA) and orthogonal partial least-squares discriminant analysis (OPLS-DA), was used to classify the Elian No. 5 and Elian No. 6 samples. Variable importance for projection (VIP) values, fold change (FC), and *p* value were used to screen the dataset and identify potential markers. These results could help the further distinguishing and development of lotus roots.

## 2. Materials and Methods

### 2.1. Sample Collection

Hubei Province is one of the main regions where lotus root is grown and possesses a planting area of more than 9.63 × 10^8^ m^2^ [26]. Two fresh lotus root cultivars, Elian No. 5 and Elian No. 6, were harvested from Hanchuan, Jiangxia, and Honghu cities of Hubei Province during the winter of 2021 (Table 1). Collected samples were transported to our laboratory in 12 h and stored at 4 °C for no more than 24 h before the subsequent treatment.

### 2.2. Lotus Root Flours and Isolated Starch Preparation

Lotus root samples were washed with tap water to remove the attached soil. After drying the surface water, the peeled lotus roots were cut into pieces, and some of the samples were immediately stored at −20 °C for 48 h and then freeze-dried (FD5-3, Gold SIM (China), Beijing, China). Some were homogenized for starch preparation. In detail, the homogenate solution after filtration was precipitated for 1 h, and the separated residue was filtered by deionized water at least four times until the supernatant was transparent. The mixture was repeatedly precipitated for 1 h, and the residue was collected and freeze-dried after removing the surface impurities. Finally, the freeze-dried pieces and starches were ground into powder using a mill (Tube-Mill 100, IKA, Staufen, Germany) and passed through a 100-mesh sieve. Then, the lotus root flours and isolated starches were stored at −20 °C for subsequent analysis.

### 2.3. Pasting Properties Analysis

The pasting properties of lotus root flours and isolated starches were investigated with a Rapid Visco-Analyzer (RVA) (Perten, RVA4500, Macquarie Park, Australia). Samples (1.5 g) were weighed accurately, and 25 mL deionized water was added to prepare 6% (*w*/*w*) suspensions. The evaluation program was as follows: the suspensions were first held at 50 °C for 2 min, heated to 95 °C at an increase rate of 12 °C/min, held for 2.5 min, and subsequently cooled to 50 °C at the same rate. Seven pasting properties were characterized, including peak viscosity (PV), trough viscosity (TV), final viscosity (FV), breakdown viscosity (BV), setback viscosity (SV), peak time (PT), and pasting temperature (PTP). Each sample was analyzed in triplicate.

### 2.4. Texture Characteristics Analysis

The clean and fresh lotus roots were cut into 1.0 ± 0.1 cm thick slices, and then the texture characteristics of slices of raw and cooked (steamed for 1 h) lotus roots were analyzed. The texture properties, including hardness, toughness, stickiness, and chewiness, of the tested samples were compared by using a Tensipresser My Boy II texture analyzer (Taketomo Electric, Tokyo, Japan) equipped with Texture Expert software (version 2.26 E). A 1 mm puncture probe at a 15 mm distance at a pretest was used for texture detection. The pretest, test, and posttest speeds were 2.0 mm/s, 1.0 mm/s, and 5 mm/s, respectively. In two cycles, the compression deformation was 35%. Experiments on each sample were repeated five times.

Moreover, the lotus root flour and starch gels obtained from RVA experiments were stored at 4 °C for 24 h and then incubated for 2 h at room temperature to test the gel texture properties by the same texture analyzer. A two-cycle compression test was implemented with a 2.5 cm diameter flat-ended cylinder probe for a 30 mm initial height at a 2.0 mm/s pretest speed and 35% compression deformation. Experiments on each sample were tested in triplicate.

### 2.5. Metabolomics Analysis by UPLC-Q-TOF-MS

Freeze-dried lotus root flours of 40 mg were weighed and dissolved in 1.8 mL precooled 50% methanol in water. The mixtures were vortexed for 1 min and oscillated for 10 min. The suspensions were centrifuged at 15,000 rpm for 10 min at 4 °C. The supernatant was collected and mixed with 0.9 mL precooled 50% methanol in water to repeat the extraction. Then, the combined supernatants were filtered for subsequent analysis. Each sample was conducted with seven replications. Quality control (QC) samples were prepared by combining equal aliquots from all extracts of the lotus freeze-dried flour and were injected into every six specimens during the whole analysis.

Chromatographic separation was achieved on a SCIEX ExionLC AD system equipped with an ACQUITY UPLC HSS T3 column (2.1 × 100 mm, 1.8 µm) and ACQUITY UPLC BEH Amide column (2.1 × 100 mm, 1.7 µm) separately maintained at 40 ℃. The injection volume was 5 µL, and the sample plate was maintained at 4 ℃. When analyzed with the HSS T3 column, a gradient elution program consisting of mobile phases A (0.05% formic acid in water) and B (acetonitrile) was used at a flow rate of 0.4 mL/min, and the gradient program was as follows: 0–1 min, 1% B; 1–24 min, 1–30% B; 24–37 min, 30–98% B; 37–42 min, 98%; 42.1–44 min, 1% B. When analyzed with the BEH Amide column, a gradient elution program consisting of mobile phases A (25 mM NH_4_FA and 25 mM NH_4_OH in water) and B (acetonitrile) was used at a flow rate of 0.3 mL/min, and the gradient program was as follows: 0–1 min, 95% B; 1–14 min, 95–65% B; 14–16 min, 65–40% B; 16–18 min, 40%; 18.1–23 min, 95% B.

The mass spectrometric data of all samples were acquired with an information-dependent acquisition (IDA) method using the ZenoTOF 7600 mass spectrometer (AB SCIEX, Framingham, MA, USA) in electrospray ionization (ESI) positive and negative ion modes. The parameters were set as follows: the ion spray voltage floating (ISVF) was set at 5500 V for positive mode and −4500 V for negative mode, the nebulizing gas (GS1) and drying gas (GS2) were both nitrogen set at 50 psi; turbo V source temperature (TEM) was set at 500 °C; collision gas (CAD) was nitrogen set at 7 psi; curtain gas (CUR) was nitrogen set at 35 psi; mass range from 60 to 1200 Da. Mass spectral data were acquired in TOFMS-IDA-TOFMSMS mode and analyzed using SCIEX OS software (version 2.2, AB SCIEX, USA). Compound identification was performed by using empirical formula finding, natural product secondary standard spectrum library, and online database searching. In addition, a Zeno trap was employed to increase the intensity of secondary iron mass spectrometry; IDA combined with dynamic background subtraction (DBS) was used to trigger acquisition of the MS/MS information of low-level constituents.

### 2.6. Data Processing and Statistical Analysis

The results from pasting and texture properties were expressed as the mean ± standard deviation (SD). Statistically significant differences were analyzed by one-way analysis of variance (ANOVA), and the differences between the means of samples were carried out by Duncan’s test using SPSS 16.0. The MS raw data (wiff.scan files) were processed with SCIEX OS software (Version 2.1). Metabolite identification was performed based on the precise mass of the molecules (<5 ppm) and the MS/MS spectra with an in-house database. All data were normalized, centered on the mean, and divided by the square root of the standard deviation of each variable (Pareto scaling). After normalization, the data were subjected to principal component analysis (PCA) and orthogonal partial least-squares discriminant analysis (OPLS-DA) by SIMCA 13.0 (Umetrics, Umea, Sweden). The measure of fit of the Model (R^2^) and the measure of predictive ability of the Model (Q^2^) were used to evaluate the models. The variable importance in the projection (VIP) value (VIP > 1) from the OPLS-DA model contributed to the classification of metabolites. Multivariate analyses including fold change (FC, Elian No. 5/Elian No. 6) and *p*-value calculation were performed by MetaboAnalyst version 5.0 (https://www.metaboanalyst.ca).

## 3. Results and Discussion

### 3.1. Pasting Behavior of Lotus Root Flours and Starches

The pasting properties of flour and starch samples from Elian No. 5 and Elian No. 6 are presented in Table 2. Generally, the pasting viscosities, including PV, TV, BV, FV, and SV, of starch samples were 1.3~8.0 times those of the whole flours, which was consistent with several previous studies [27,28]. The differences between flours and starches were related to the presence of lipids, proteins, fibers, and lower starch in flours. Most pasting viscosities of Elian No. 5 flours were significantly higher (*p* < 0.05) than those of Elian No. 6 flours, and the coefficients of variation were all over 30%. Notably, the BVs of Elian No. 5 flours and starches were both higher than those of Elian No. 6 flours and starches, indicating that Elian No. 5 possessed a lower ability to withstand shear stress and heating during cooking [29,30]. The SVs of the flours and starches of Elian No. 6 were both lower than those of Elian No. 5; thus, Elian No. 5 might have a remarkable tendency to retrograde compared to Elian No. 6 during the process. These results further confirmed the difference between the Elian No. 5 and Elian No. 6 cultivars. The pasting property trends of Elian No. 5 from the three planting areas were similar to those of Elian No. 6.

### 3.2. Texture Properties of Lotus Root Slices and Gels

**Figure 1:**
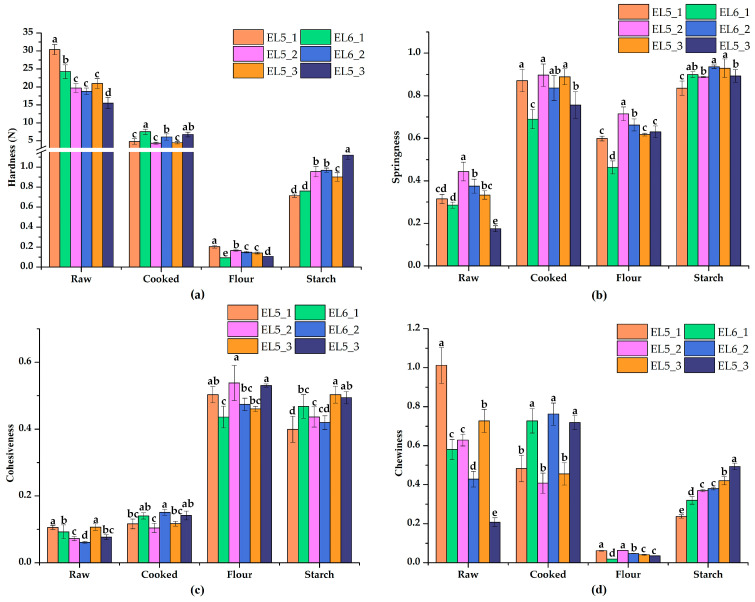
Texture profile analysis of lotus root samples processed in different ways. (**a**) Hardness; (**b**) Springiness; (**c**) Cohesiveness; (**d**) Chewiness. Error bars represent means ± SDs. Raw—raw lotus root slices; Cooked—lotus root slices steamed for 1 h; Flour—gel of whole lotus root powder; Starch—gel of isolated lotus root starch. Different letters in the same group represent significant differences (*p* < 0.05).

The results of the texture properties of raw and cooked lotus root slices, as well as the flour and starch gels, are presented in Figure 1. It was evident that the hardness of the raw slices and flour gel from Elian No. 5 were higher than that of Elian No. 6, but the hardness of cooked lotus root slices and starch gels were significantly lower (*p* < 0.05, Figure 1a). A similar trend was observed in the chewiness value, a parameter that was related to hardness (Figure 1d). These results further proved that the texture of Elian No. 5 became farinose, while Elian No. 6 remained crisp during cooking, since the hardness of Elian No. 5 dropped more. It is well known that cooking heat treatment results in the decomposition of intercellular mucus and softening of the texture [31]. Liu et al. [32] indicated that cell wall polyacrylamide, especially the ratio of chelate-soluble fraction to alcohol-insoluble residue, was the major factor affecting the texture of lotus root after cooking. Beyond that, our results showed that the retrogradation behavior of starch also affected hardness, while the hardness of the flour gel of Elian No. 5 was still slightly higher than that of Elian No. 6, and the additional analysis of composition differences between flours and isolated starches may provide a more detailed explanation [33,34]. The springiness of raw and cooked slices from Elian No. 5 was higher than that of Elian No. 6, but the springiness of flour and starch samples had no obvious regularity (Figure 1b). The cohesiveness (Figure 1c) of raw and cooked slices was quite the opposite between Elian No. 5 and Elian No. 6, while it was disorderly in flour and starch gels. The texture properties strongly suggested the opposite texture of Elian No. 5 and Elian No. 6, but this feature was consistent in the three planting areas.

### 3.3. Interpretation of Zeno-TOF-MS Mass Spectra and Metabolite Profiles of Lotus Root Samples

Accurate identification of metabolites is particularly important in metabolomics studies, which are dependent on high-quality secondary mass spectrometry. In this study, a Zeno trap performed on a ZenoTOF™ 7600 system [24] was first implemented for the systematic comparison of the metabolites of lotus root cultivars with distinct textures. The potential compounds were identified using an in-house MS/MS high-resolution library containing over 700 common metabolites. The metabolites with mass error < 5 ppm, isotope difference < 10%, and library hit score > 80 were classified as identified. Compared with the Zeno trap-off model, the intensity of the spectrum from the Zeno trap-on model increased 5 to 20 times (Figure S1a); meanwhile, a higher matching score of the secondary mass spectrometry library was obtained with a low injection concentration (Figure S1b). Thus, the Zeno trap contributed to the accurate identification of samples with low-abundance metabolites. Based on this method, the metabolite profile of freeze-dried lotus root samples was represented, and a total of 124 and 64 kinds of compounds were identified by positive and negative ion modes, respectively (Table S1). Consistent with previous findings, lotus roots were rich in bioactive substances [8,35]. In this study, all identified compounds mainly included 3 kinds of alkaloids, 36 kinds of amino acids and their derivatives, 4 kinds of coumarins, 3 kinds of flavone glycosides, 26 kinds of flavonoids, 2 kinds of isoquinolines, 1 kind of lignan, 23 kinds of lipids, 13 kinds of nucleotides, 10 kinds of organic acids, 9 kinds of polyphenols, 24 kinds of saccharides and their derivatives, 7 kinds of vitamins, 3 kinds of terpenoids, and 24 other compounds.

### 3.4. Principal Component Analysis of Metabolites in Lotus Roots

**Figure 2:**
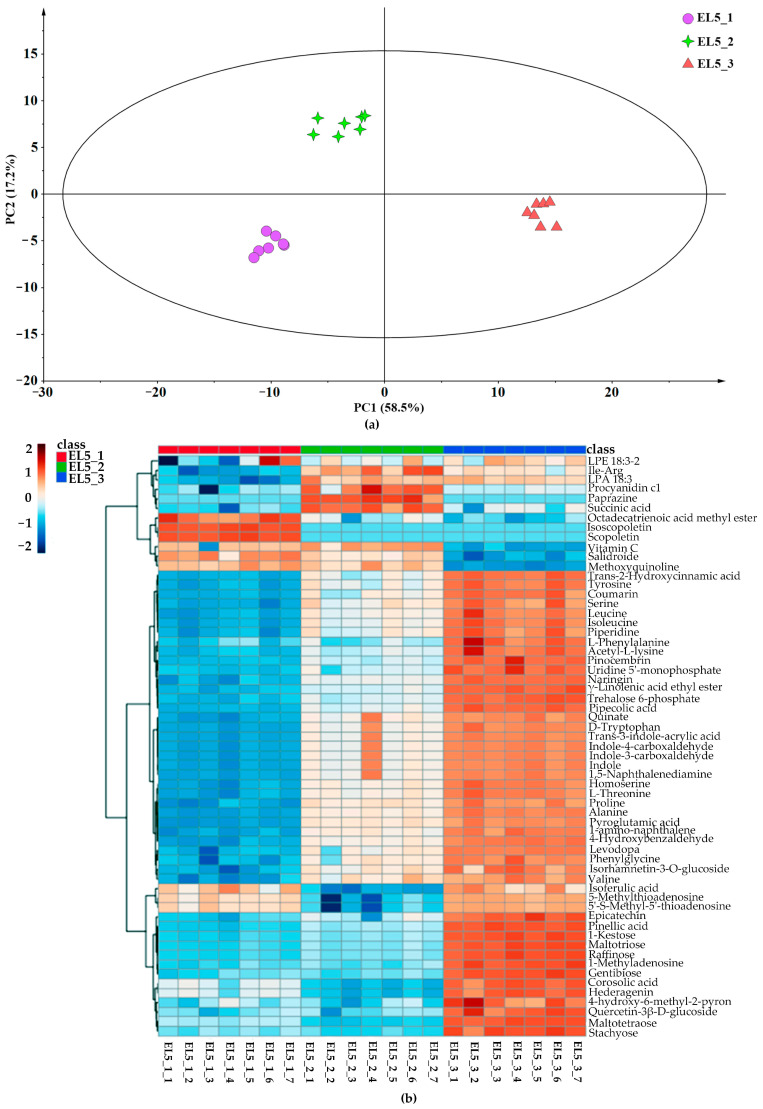

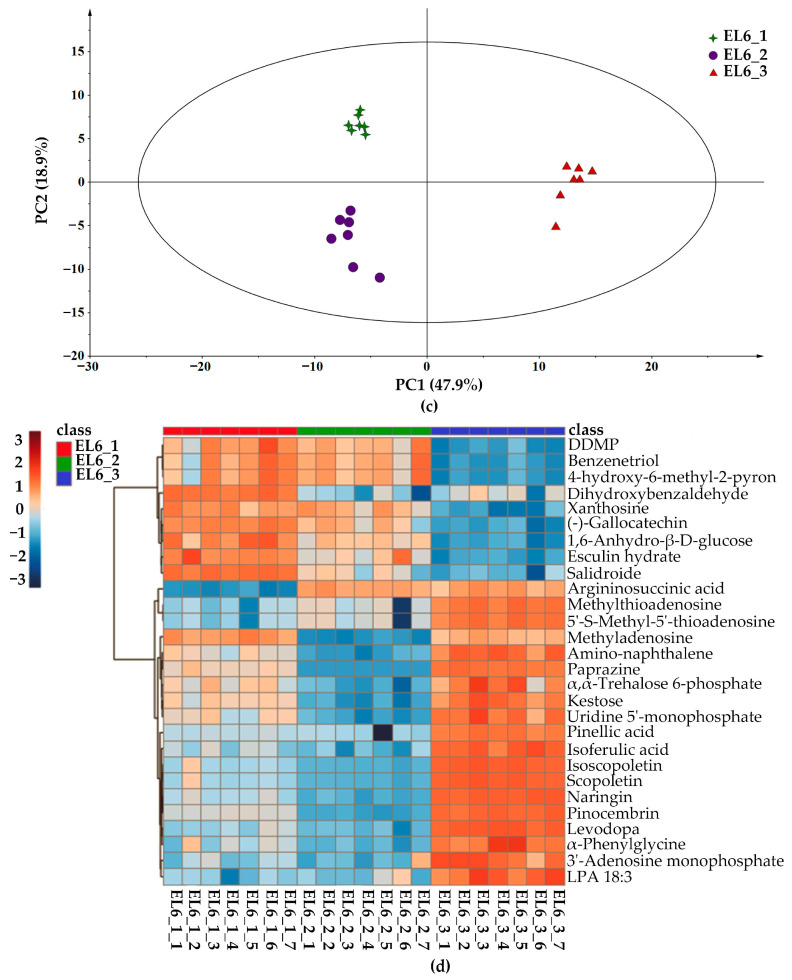
Principal component analysis and differential metabolite analysis of lotus root samples from different geographical origins. (**a**). PCA score plot of Elian No. 5 from Hanchuan (EL5_1, marked as light green star), Jiangxia (EL5_2, marked as light purple dots), and Honghu (EL5_3, marked as light red triangle). (**b**). Heatmap according to the differential metabolites of Elian No. 5 from three geographical origins. (**c**). PCA score plot of Elian No. 6 from Hanchuan (EL6_1, marked as dark green star), Jiangxia (EL6_2, marked as dark purple dots), and Honghu (EL6_3, marked as dark red triangle). (**d**). Heatmap according to the differential metabolites of Elian No. 6 from three geographical origins.

Unsupervised PCA models were constructed to illustrate the differences in the metabolites of different lotus root species from three geographical origins, Hanchuan, Jiangxia, and Honghu cities. The PCA score plot for all lotus root samples and QC samples (Figure S2) could be clearly distinguished with a total variance of 60.9%, in which PC1 and PC2 explained 43.9% and 17% of the total variance, respectively. It was obvious that the metabolites of the same lotus root cultivar from the three producing areas were significantly different and could be effectively distinguished. As shown in the PCA score plot (Figure 2a) obtained from the metabolites of Elian No. 5, the principal components explained 58.5% and 17.2% of the total variation for three geographical origins. Then, a cluster analysis was performed to investigate the relative abundances of identified metabolites, as visualized using a heatmap (Figure 2b). The metabolites identified from different origins were clearly divided into 3 groups. There were 61 differential metabolites identified with VIP > 1, including isoscopoletin, naringin, vitamin C, and coumarin. In addition, the relative abundance of differential metabolites in lotus samples from Honghu was higher than those from Hanchuan and Jiangxia. Similarly, the PCA score plot obtained from the metabolites of Elian No. 6 is presented in Figure 2c, and the principal components explained 47.9% and 18.9% of the total variation for the three geographical origins. The heatmap (Figure 2d) showed only 28 differential metabolites among the three groups. Interestingly, gallocatechin, esculin hydrate, and salidroside were richer in Elian No. 6 from Hanchuan and Jiangxia, while some other functional compounds, such as argininosuccinic acid, levodopa, isoferulic acid, and pinocembrin, possessed significantly higher abundance in the Honghu group. Recently, non-target approaches for food authenticity have been widely used [36], LC-MS-based metabolomics was effective for the authentication of narrow-geographic samples [37], while the geographical origin analysis of lotus root was limited [38]. This study further indicated that the metabolism of lotus roots was significantly affected by growing regions [39], but there is very little known about the application of most of these metabolites. Future studies focused on these functional metabolites could help the discrimination of lotus roots, as well as boost the consumption of lotus root products with regional characteristics.

### 3.5. Orthogonal Projections to Latent Structures-Discriminant Analysis of the Metabolites in Lotus Roots

**Figure 3:**
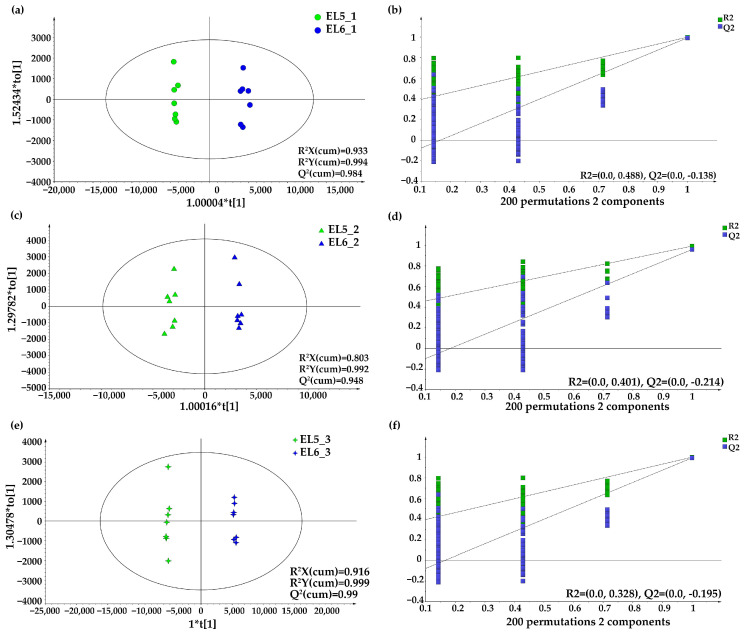
OPLS-DA score plots and Model 200 permutation tests of different lotus root species. (**a**,**b**): OPLS-DA score plot (**a**) and Model 200 permutation tests (**b**) of Elian No. 5 and Elian No. 6 samples from Hanchuan. Green dots: Elian No. 5 samples from Hanchuan (EL5_1). Blue dots: Elian No. 6 samples from Hanchuan (EL6_1). Green square: R^2^, the fit ability. Blue square: Q^2^, the predictive ability. (**c**,**d**): OPLS-DA score plot (**c**) and Model 200 permutation tests (**d**) of Elian No. 5 and Elian No. 6 samples from Jiangxia. Green triangles: Elian No. 5 samples from Jiangxia (EL5_2). Blue triangles: Elian No. 6 samples from Jiangxia (EL6_2). (**e**,**f**): OPLS-DA score plot (**e**) and Model 200 permutation tests (**f**) of Elian No. 5 and Elian No. 6 samples from Honghu. Green stars: Elian No. 5 samples from Honghu (EL5_3). Blue stars: Elian No. 6 samples from Honghu (EL6_3).

Orthogonal projections to latent structures-discriminant analysis (OPLS-DA) was performed to distinguish Elian No. 5 and Elian No. 6 cultivars (Figure 3) because of its powerful ability to classify samples that contain only two groups [40]. In this study, the OPLS-DA models after the pretreatment method (Par-scaling) revealed the best classification result [41]. Generally, three OPLS-DA score plots of the tested samples from three regions were sufficiently fit and predictive, since both of them had satisfied the conditions that the R^2^Y(cum) and Q^2^(cum) values were greater than 0.5 [42]. Specifically, R^2^Y(cum) = 0.994 and Q^2^(cum) = 0.984 in the extracts of Elian No. 5 and Elian No. 6 from Hanchuan (Figure 3a), R^2^Y(cum) = 0.992 and Q^2^(cum) = 0.948 in the extracts of Elian No. 5 and Elian No. 6 from Jiangxia (Figure 3c), and R^2^Y(cum) = 0.999 and Q^2^(cum) = 0.99 in the extracts of Elian No. 5 and Elian No. 6 from Honghu (Figure 3e) indicated a significant difference between the two lotus root cultivars. Moreover, 200 permutation test results showed that the y-intercepts of the three models were all less than 0.05 for the Q^2^ intercept [43]. As shown in Figure 3, the Q2 values were −0.138, −0.214, and −0.195 for the above models, further demonstrating the reliability of the three models without any overlap.

To further compare the differences in metabolites between the two types of lotus root cultivars, the variables with VIP > 1, *p* value < 0.05, FC value >2 or <0.5 were used to identify potential markers in each region [41]. First, 22 significantly differential metabolites were identified between the Elian No. 5 and Elian No. 6 samples from Hanchuan (Table 3a). Obviously, the relative amounts of 6-methoxyquinoline and serotonin in Elian No. 5 were higher than those in Elian No. 6, but the remaining 22 metabolites, including pipecolic acid, coumarin, scopoletin, and paprazine, were lower. Second, 12 significantly differential metabolites were identified between Elian No. 5 and Elian No. 6 samples from Jiangxia (Table 3b). The relative amounts of naringin, pinocembrin, α-phenylglycine, 6-methoxyquinoline, and levodopa in Elian No. 5 were higher, while those of isoscopoletin, paprazine, scopoletin, 5′-s-methyl-5′-thioadenosine, 5-methylthioadenosine, isoferulic acid, and γ-aminobutyric acid in Elian No. 5 were lower. Third, 15 significantly differential metabolites were identified between the Elian No. 5 and Elian No. 6 samples from Honghu (Table 3c). Unlike in the Hanchuan and Jiangxia groups, the levels of 10 differential metabolites, including hederagenin, corosolic acid, and esculin hydratein, in Elian No. 5 were higher than those in Elian No. 6, and only four compounds (isoscopoletin, paprazine, scopoletin, and tyramine) were lower. Notably, the levels of isoscopoletin, scopoletin, and paprazine in all tested Elian No. 5 samples were significantly lower than those in all tested Elian No. 6 samples, indicating their potential for discriminating Elian No. 5 and Elian No. 6 cultivars, as well as farinose and crisp lotus roots. Many studies have comparatively analyzed the metabolites of different lotus root cultivars or different parts of the lotus [21,35,44], while the application for the classification of different lotus genotypes is immature, which need to be investigated in future research.

## 4. Conclusions

Pasting and texture properties were analyzed between two farinose and crisp lotus root cultivars, Elian No. 5 and Elian No. 6, and the results strongly proved the opposite textures of Elian No. 5 and Elian No. 6, but this feature was consistent in different planting areas. For the first time, untargeted metabolite profiling of Elian No. 5 and Elian No. 6 from three geographical regions was performed by UPLC-Q-TOF-MS with a ZenoTOF™ 7600 system equipped with a Zeno trap approach. In total, 188 metabolites were identified from the chemical database, and these metabolites varied significantly among lotus roots from different cultivars and origins according to multivariate analysis. Isoscopoletin, scopoletin, and paprazine showed potential for discriminating between the Elian No. 5 and Elian No. 6 cultivars. In conclusion, the UPLC-Q-TOF-MS method described in this study can be useful for lotus root classification. Further research based on a large sample set including more cultivars and crop seasons must be conducted to broaden the knowledge and support further utilization of lotus roots in food industries.

## Figures and Tables

**Table 1:** Information for tested lotus root cultivars.

Sample No.	Cultivars	Appearance	Sown/Harvest Time	Harvest City
EL5-1	Elian No.5	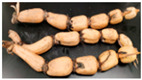	Mid-Mar/Mid-Nov	Hanchuan, 30°49′ N, 113°51′ E
EL6-1	Elian No.6	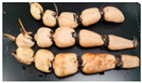		
EL5-2	Elian No.5	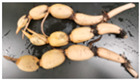	Early-Apr/Late-Nov	Jiangxia, 30°23′ N, 114°14′ E
EL6-2	Elian No.6	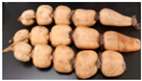		
EL5-3	Elian No.5	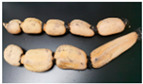	Early-Apr/Mid-Dec	Honghu, 30°03′ N, 113°39′ E
EL6-3	Elian No.6	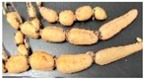		

**Table 2:** Pasting properties (mean ± SD, *n* = 3) of lotus root flours and isolated starches.

Pasting Properties		Samples					
		EL5-1	EL6-1	EL5-2	EL6-2	EL5-3	EL6-3
PV (cP)	Flour	430 ± 6.6 ^a^	207 ± 2.5 ^e^	333 ± 1.2 ^c^	305 ± 1.7 ^d^	340 ± 3.8 ^b^	180 ± 1.5 ^f^
	Starch	1164 ± 21 ^c^	1265 ± 7.5 ^b^	1258 ± 14 ^b^	1322 ± 9.2 ^a^	1273 ± 9.9 ^b^	1272 ± 15 ^b^
TV (cP)	Flour	328 ± 5.5 ^a^	152 ± 2.1 ^e^	271 ± 3.1 ^c^	248 ± 1.0 ^d^	283 ± 3.5 ^b^	140 ± 2.3 ^f^
	Starch	1035 ± 11 ^e^	1197 ± 8.5 ^a^	1040 ± 9.1 ^e^	1137 ± 8.6 ^c^	1062 ± 4.7 ^d^	1171 ± 8.4 ^b^
BV (cP)	Flour	102 ± 2.1 ^a^	56 ± 0.6 ^c^	62 ± 2.0 ^b^	57 ± 1.0 ^c^	56 ± 1.2 ^c^	40 ± 1.0 ^d^
	Starch	138 ± 13 ^c^	74 ± 3.2 ^e^	227 ± 6.6 ^a^	179 ± 5.0 ^b^	218 ± 4.5 ^a^	106 ± 2.5 ^d^
FV (cP)	Flour	403 ± 5.9 ^a^	177 ± 2.5 ^e^	332 ± 4.6 ^c^	305 ± 2.0 ^d^	345 ± 4.2 ^b^	167 ± 2.5 ^f^
	Starch	1222 ± 4.5 ^c^	1352 ± 9.1 ^a^	1238 ± 6.0 ^bc^	1335 ± 4.0 ^a^	1244 ± 3.5 ^b^	1346 ± 19 ^a^
SV (cP)	Flour	74 ± 0.6 ^a^	25 ± 1.0 ^d^	61 ± 1.5 ^b^	57 ± 2.6 ^c^	62 ± 1.0 ^b^	27 ± 1.0 ^d^
	Starch	185 ± 14 ^b^	164 ± 2.5 ^c^	205 ± 3.8 ^a^	199 ± 4.7 ^a^	182 ± 5.0 ^b^	178 ± 8.2 ^b^
PT (cP)	Flour	5.93 ± 0.06 ^abc^	5.95 ± 0.04 ^ab^	5.91 ± 0.08 ^bc^	6.05 ± 0.04 ^a^	6.04 ± 0.10 ^a^	5.82 ± 0.04 ^c^
	Starch	7.00 ± 0.00 ^a^	6.99 ± 0.02 ^a^	6.83 ± 0.17 ^b^	7.01 ± 0.02 ^a^	6.99 ± 0.02 ^a^	7.00 ± 0.00 ^a^
PTP (°C)	Flour	78 ± 0.4 ^a^	74 ± 0.1 ^b^	78 ± 0.0 ^a^	74 ± 0.0 ^b^	74 ± 0.1 ^b^	78 ± 0.5 ^a^
	Starch	75 ± 0.4 ^a^	74 ± 0.5 ^b^	75 ± 0.0 ^a^	73 ± 0.0 ^bc^	73 ± 0.4 ^c^	73 ± 0.9 ^bc^

Different letters in the same line represent significant differences (*p* < 0.05). PV, peak viscosity; TV, through viscosity; BV, breakdown viscosity; FV, final viscosity; SV, setback viscosity; PT, peak time; PTP, pasting temperature.

**Table 3:** The identified potential markers in Elian No.5 and Elian No.6 samples from Hanchuan (**a**), Jiangxia (**b**), and Honghu (**c**).

**(a)**								
**Species**	**Metabolites**	**VIP**	**Fold Change**	***p*-Value**	**Formula**	**Adduct Type**	***m*/*z***	**Mass Error (ppm)**
Amino acid derivative	Pipecolic acid	1.26	0.46	1.11 × 10^−15^	C_6_H_11_NO_2_	[M+H]^+^	130.0863	0
Amino acid	Tryptophan	1.25	0.34	3.43 × 10^−15^	C_11_H_12_N_2_O_2_	[M+H]^+^	205.0968	−1.6
	Leucine	1.25	0.49	3.32 × 10^−11^	C_6_H_13_NO_2_	[M+H]^+^	132.1017	−1.4
	Tyrosine	1.25	0.47	3.38 × 10^−11^	C_9_H_11_NO_3_	[M+H]^+^	182.0811	−0.4
Alkaloid	Scopoletin	1.23	0.28	8.21 × 10^−9^	C_10_H_8_O_4_	[M+H]^+^	193.0495	0
Lipids	Glycerophosphocholine	1.24	0.44	5.41 × 10^−11^	C_8_H_20_NO_6_P	[M+H]^+^	258.1102	0.4
	Phosphocholine	1.15	0.44	5.98 × 10^−6^	C_5_H_14_NO_4_P	[M+H]^+^	184.0733	−0.4
Nucleotide	Uridine 5′-monophosphate	1.24	0.47	2.40 × 10^−10^	C_9_H_13_N_2_O_9_P	[M−H]^−^	323.0280	−1.7
Polyphenol	Trans-2-Hydroxycinnamic acid	1.26	0.46	2.86 × 10^−13^	C_9_H_8_O_3_	[M+H]^+^	165.0546	−0.4
	4-Hydroxybenzaldehyde	1.25	0.49	2.60 × 10^−12^	C_7_H_6_O_2_	[M+H]^+^	123.0440	−0.8
	Coumarin	1.24	0.47	2.57 × 10^−10^	C_9_H_6_O_2_	[M+H]^+^	147.0439	−0.8
	Isoscopoletin	1.23	0.28	8.21 × 10^−9^	C_10_H_8_O_4_	[M+H]^+^	193.0495	0
Quinolines	Quinoline	1.25	0.41	4.57 × 10^−12^	C_9_H_7_N	[M+H]^+^	130.0651	−0.3
	6-Methoxyquinoline	1.22	2.58	4.12 × 10^−10^	C_10_H_9_NO	[M+H]^+^	160.0757	−0.2
Other	Paprazine	1.26	1.55 × 10^−6^	9.61 × 10^−22^	C_17_H_17_NO_3_	[M+H]^+^	284.1277	−1.4
	1,5-Naphthalenediamine	1.26	0.37	5.12 × 10^−15^	C_10_H_10_N_2_	[M+H]^+^	159.0915	−1
	Trans-3-indole-acrylic acid	1.25	0.37	5.06 × 10^−14^	C_11_H_9_NO_2_	[M+H]^+^	188.0706	0.2
	Indole	1.25	0.36	3.19 × 10^−14^	C_8_H_7_N	[M+H]^+^	118.0650	−1.2
	Indole-4-carboxaldehyde	1.25	0.37	1.09 × 10^−13^	C_9_H_7_NO	[M+H]^+^	146.0599	−1
	1-amino-naphthalene	1.25	0.27	5.04 × 10^−12^	C_10_H_9_N	[M+H]^+^	144.0806	−1
	4-oxo-5α-(2Z)-pentenyl-2-cyclopentene-1α-octanoic acid	1.22	0.49	2.46 × 10^−8^	C_18_H_28_O_3_	[M+H]^+^	293.2108	−1.2
	Serotonin	1.03	6.31	1.98 × 10^−4^	C_10_H_12_N_2_O	[M+H]^+^	177.1021	−0.8
**(b)**								
**Species**	**Metabolites**	**VIP**	**Fold Change**	***p*-Value**	**Formula**	**Adduct Type**	***m*/*z***	**Mass Error (ppm)**
Amino acid derivative	Carboxyethyl-γ-aminobutyric acid	1.27	0.46	1.53 × 10^−4^	C_7_H_13_NO_4_	[M+H]^+^	176.0918	0.1
	α-Phenylglycine	1.35	2.64	3.10 × 10^−5^	C_8_H_9_NO_2_	[M+H]^+^	152.0704	−1.6
	Levodopa	1.22	2.01	8.00 × 10^−4^	C_9_H_11_NO_4_	[M+H]^+^	198.0759	−0.9
Alkaloid	Scopoletin	1.51	0.1	3.97 × 10^−18^	C_10_H_8_O_4_	[M+H]^+^	193.0495	0
Flavonoids	Naringin	1.49	6.36	2.93 × 10^−11^	C_27_H_32_O_14_	[M+H]^+^	581.1858	−1.1
	Pinocembrin	1.5	3.83	3.43 × 10^−11^	C_15_H_12_O_4_	[M+H]^+^	257.0806	−0.7
Nucleotide	5-Methylthioadenosine	1.07	0.45	3.25 × 10^−3^	C_11_H_15_N_5_O_3_S	[M+H]^+^	298.0969	0.3
Polyphenol	Isoscopoletin	1.51	7.15 × 10^−7^	1.67 × 10^−27^	C_10_H_8_O_4_	[M+H]^+^	193.0495	0
	Isoferulic acid	1.42	0.45	4.82 × 10^−7^	C_10_H_10_O_4_	[M−H]^-^	193.0506	−0.4
Quinolines	6-Methoxyquinoline	1.35	2.08	7.27 × 10^−6^	C_10_H_9_NO	[M+H]^+^	160.0757	−0.2
Other	Paprazine	1.51	5.04 × 10^5^	1.35 × 10^−20^	C_17_H_17_NO_3_	[M+H]^+^	284.1277	−1.4
**(c)**								
**Species**	**Metabolites**	**VIP**	**Fold Change**	***p*-Value**	**Formula**	**Adduct Type**	***m*/*z***	**Mass Error (ppm)**
Alkaloid	Scopoletin	1.33	0.01	2.21 × 10^−22^	C_10_H_8_O_4_	[M+H]^+^	193.0495	0
Flavonoids	Hederagenin	1.33	2.65	3.58 × 10^−14^	C_30_H_48_O_4_	[M−H]^-^	471.3475	−0.9
Lipids	γ-Linolenic acid ethyl ester	1.3	2.43	3.29 × 10^−9^	C_20_H_34_O_2_	[M+H]^+^	307.2626	−1.9
	γ-Linolenic Acid	1.3	2.21	3.25 × 10^−9^	C_18_H_30_O_2_	[M−H]^-^	277.2168	−2
Nucleotide	Xanthosine	1.26	2.29	3.29 × 10^−7^	C_10_H_12_N_4_O_6_	[M−H]^-^	283.0678	−2.1
Polyphenol	Isoscopoletin	1.33	8.92 × 10^−8^	3.69 × 10^−29^	C_10_H_8_O_4_	[M+H]^+^	193.0495	0
	Isoferulic acid	1.3	0.41	2.20 × 10^−9^	C_10_H_10_O_4_	[M−H]^-^	193.0506	−0.4
	Esculin hydrate	1.26	2.59	2.26 × 10^−7^	C_15_H_16_O_9_	[M+H]^+^	341.0866	−0.4
	Scopolin	1.32	2.47	3.86 × 10^−11^	C_16_H_18_O_9_	[M+H]^+^	355.1020	−1.1
Saccharides	N-Acetyl-β-D-mannosamine	1.03	2.87	1.22 × 10^−3^	C_8_H_15_NO_6_	[M+H]^+^	222.0969	−1.3
	N-Acetyl-D-glucosamine	1.18	2.58	3.56 × 10^−5^	C_8_H_15_NO_6_	[M+H]^+^	222.0969	−1.3
Terpenoid	Corosolic acid	1.33	2.65	3.58 × 10^−14^	C_30_H_48_O_4_	[M−H]^-^	471.3475	−0.9
Other	Tyramine	1.26	0.27	2.36 × 10^−7^	C_8_H_11_NO	[M+H]^+^	138.0905	−6.3
	Paprazine	1.33	4.37 × 10^−7^	1.07 × 10^−27^	C_17_H_17_NO_3_	[M+H]^+^	284.1277	−1.4
	L-Carnitine	1.33	2.15	5.92 × 10^−13^	C_7_H_15_NO_3_	[M+H]^+^	162.1123	−0.8

## Data Availability

The data used to support the findings of this study can be made available by the corresponding author upon request.

## Supplementary Materials

The following supporting information can be downloaded at: https://www.mdpi.com/article/10.3390/foods12132493/s1, Figure S1: the intensity (**a**) and matching score (**b**) of spectrum from Zeno trap-on and Zeno trap-off model; Figure S2: the PCA score plot for all lotus root samples and QC samples; Table S1: the identified metabolites in lotus root.
