# Supplementary figures and images for: Comparative Analysis of the Physicochemical Properties and Metabolites of Farinose and Crisp Lotus Roots (Nelumbo nucifera Gaertn.) with Different Geographical Origins
Source: Foods. 2023 Jun 27;12(13):2493. doi: 10.3390/foods12132493 (PMC10340570; doi:10.3390/foods12132493)

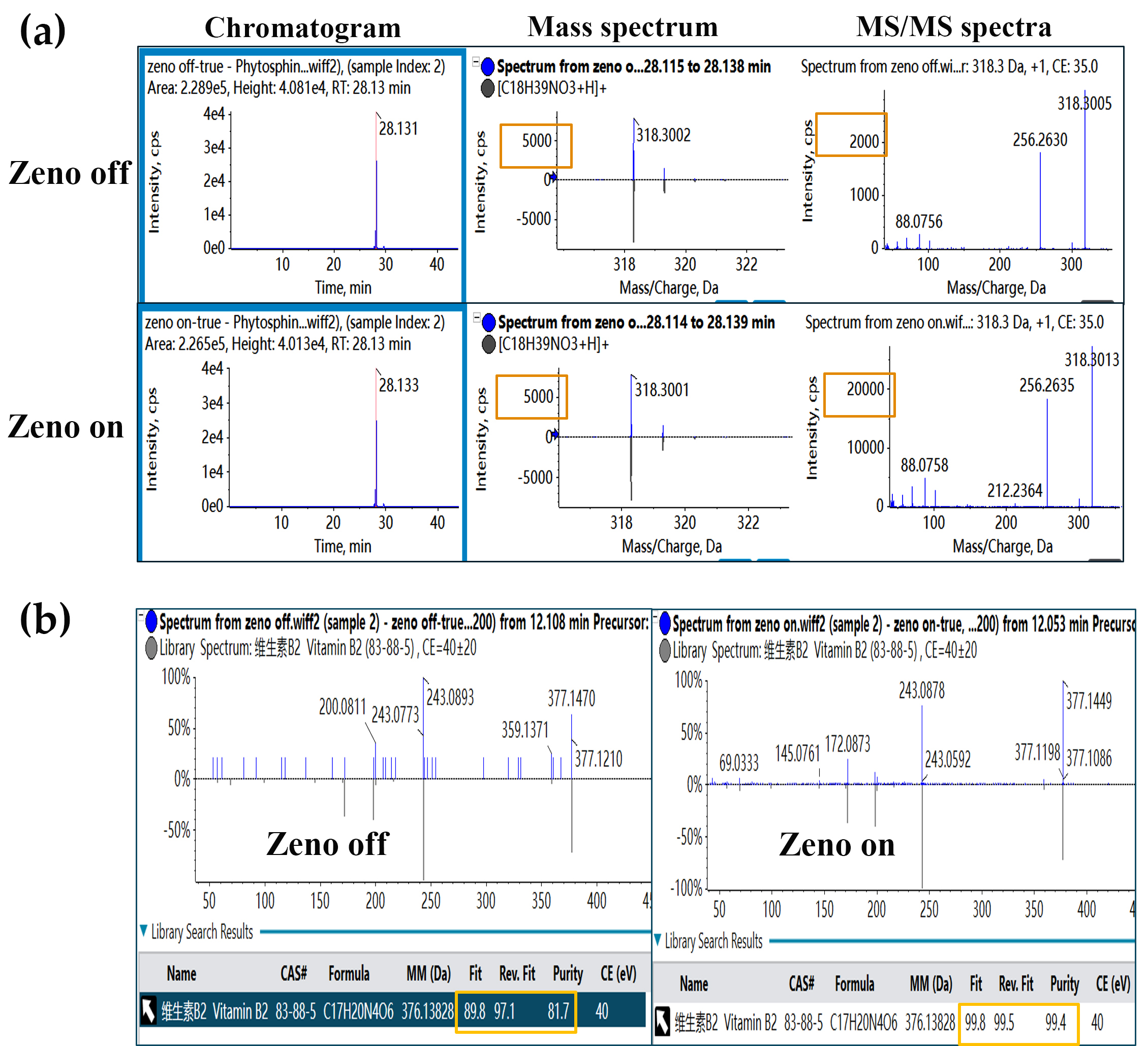

Supplement: Supplementary file 1 [file foods-12-02493-s001.zip › Supplementary materials/Figure S1.jpg]

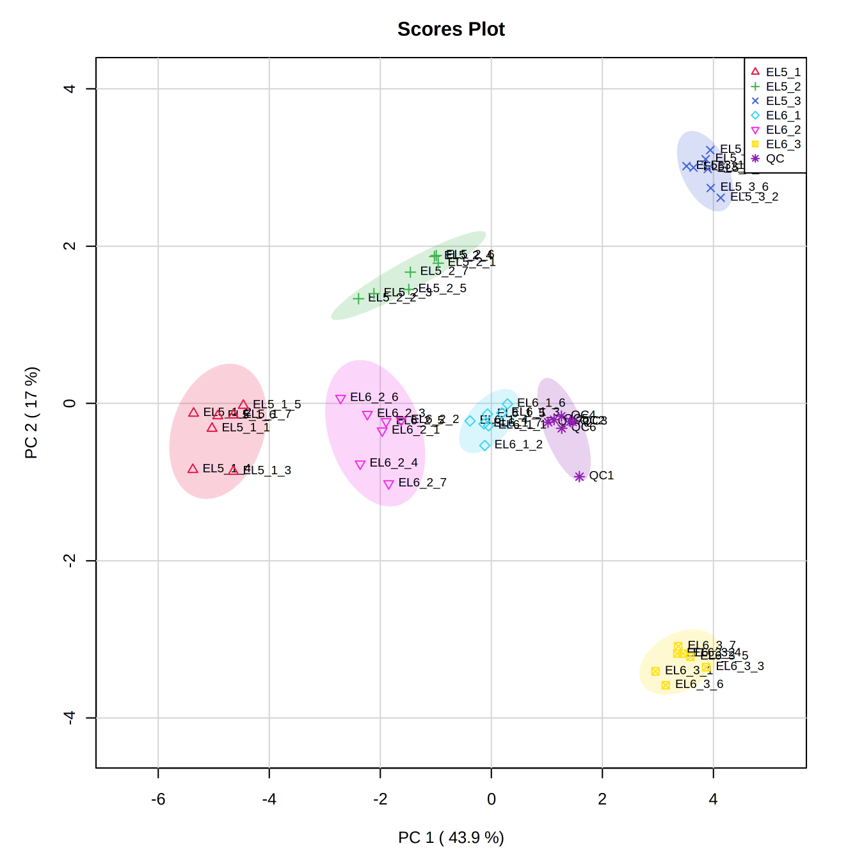

Supplement: Supplementary file 1 [file foods-12-02493-s001.zip › Supplementary materials/Figure S2.tif]
